# Placental co-transcriptional activator Vestigial-like 1 (VGLL1) drives tumorigenesis via increasing transcription of proliferation and invasion genes

**DOI:** 10.3389/fonc.2024.1403052

**Published:** 2024-06-07

**Authors:** Heather M. Sonnemann, Barbara Pazdrak, Barbara Nassif, Yimo Sun, Lama Elzohary, Amjad H. Talukder, Arjun S. Katailiha, Krishna Bhat, Gregory Lizée

**Affiliations:** ^1^ University of Texas MD Anderson Cancer Center, UTHealth Graduate School of Biomedical Sciences, Houston, TX, United States; ^2^ Department of Melanoma Medical Oncology, UT MD Anderson Cancer Center, Houston, TX, United States; ^3^ Department of Translational Molecular Pathology, UT MD Anderson Cancer Center, Houston, TX, United States; ^4^ Department of Immunology, UT MD Anderson Cancer Center, Houston, TX, United States

**Keywords:** pancreatic cancer, breast cancer, migration and invasion, mechanism of tumor progression, mechanism of transcription, cell proliferation

## Abstract

**Introduction:**

Vestigial-like 1 (VGLL1) is a co-transcriptional activator that binds to TEA domain-containing transcription factors (TEADs). Its expression is upregulated in a variety of aggressive cancer types, including pancreatic and basal-like breast cancer, and increased transcription of VGLL1 is strongly correlated with poor prognosis and decreased overall patient survival. In normal tissues, VGLL1 is most highly expressed within placental trophoblast cells, which share the common attributes of rapid cellular proliferation and invasion with tumor cells. The impact of VGLL1 in cancer has not been fully elucidated and no VGLL1-targeted therapy currently exists.

**Methods:**

The aim of this study was to evaluate the cellular function and downstream genomic targets of VGLL1 in placental, pancreatic, and breast cancer cells. Functional assays were employed to assess the role of VGLL1 in cellular invasion and proliferation, and ChIP-seq and RNAseq assays were performed to identify VGLL1 target genes and potential impact using pathway analysis.

**Results:**

ChIP-seq analysis identified eight transcription factors with a VGLL1-binding motif that were common between all three cell types, including TEAD1-4, AP-1, and GATA6, and revealed ~3,000 shared genes with which VGLL1 interacts. Furthermore, increased VGLL1 expression led to an enhancement of cell invasion and proliferation, which was supported by RNAseq analysis showing transcriptional changes in several genes known to be involved in these processes.

**Discussion:**

This work expands our mechanistic understanding of VGLL1 function in tumor cells and provides a strong rationale for developing VGLL1-targeted therapies for treating cancer patients.

## Introduction

Vestigial-like 1 (VGLL1) is a mammalian co-transcriptional activator that has been shown to play an important role in placental development and is also highly expressed in various cancer types. It was first discovered as a co-transcriptional activator in drosophila called Vestigial (vg), where it functions as a key master regulator of wing development (1, 2). Substitution of drosophila vg with the human ortholog VGLL1 (previously designated tondu) rescued wing development, underscoring the high level of conservation of this gene and its encoded protein (2). Studies over the past decade have begun illuminating the role of mammalian VGLL1 and have focused largely on two broad areas of developmental biology: normal placental development and tumorigenesis. VGLL1 is expressed at a low to negligible level in most normal tissues but demonstrates by far the highest expression in placental trophoblasts (3–5). These cells are highly proliferative and invasive, attributes that are critical for embryonic implantation (6, 7). Although little is known about the precise role VGLL1 plays in placental development, aberrantly elevated expression of this gene has also been observed in several aggressive cancer types. Pancreatic, triple negative breast, gastric, and HPV-related cancers such as ovarian cancer show the highest levels of VGLL1 mRNA expression, and increased transcription of VGLL1 is strongly correlated with poor prognosis and lower overall patient survival (3, 4, 8–11).

Crucial clues about the function of VGLL1 came from molecular studies designed to understand the structure-function relationship between TEA domain-containing transcription factors (TEADs) and different co-transcriptional activators. These studies revealed that the binding of VGLL1 to TEADs showed high structural similarity to that of co-transcriptional activators in the Hippo pathway, Yes-associated protein 1 (YAP1), and WW Domain containing transcription regulator 1 (WWTR1/TAZ) (2, 12). Since the hippo pathway has been strongly implicated in the tumorigenesis of many cancer types, this study shed important light on the potential functional role of VGLL1 in cancer (13). Furthermore, YAP1/TAZ both have well-characterized roles in the development of many normal tissues, including cytotrophoblast differentiation in the placenta (7). The intriguing relationship between tumorigenesis and placental development has been widely speculated for several decades (14, 15). However, recent evidence is emerging that VGLL1 may provide a crucial mechanistic link between these two processes.

Cancer cells and placental trophoblasts share at least three cardinal attributes: rapid cellular proliferation, tissue invasion, and the induction of immune suppression (6, 16, 17). Therefore, in the current study we investigated the effect of VGLL1 on cell proliferation and invasion by downregulation or upregulation of its expression levels in cancer cells originating from pancreas, breast, and placenta using siRNA knockdown or retrovirus overexpression systems. In addition, we performed RNA sequencing analysis of these tumor cells to evaluate transcriptional changes associated with regulating VGLL1 protein expression. Finally, ChIP-seq analysis was performed to identify and compare the chromosomal loci with which VGLL1 interacts in the three different cancer cell types. These studies confirm a key role for VGLL1 in driving cell proliferation and invasion by promoting the expression of specific genes involved in these processes and provide novel mechanistic insights into how VGLL1 may contribute to tumor aggressiveness and poor clinical outcomes in cancer patients.

## Methods

### Cell culture

Human pancreatic cell lines PANC1 (cat#CRL-1469, RRID: CVCL_0480), PANC10.05 (RRID: CVCL_1639, cat#CRL-2547), Capan1 (RRID: CVCL_0237, cat#HTB-79), Capan2 (RRID: CVCL_0026, cat#HTB-80), SU8686 (RRID: CVCL_3881, cat#CRL-1837) and human breast cancer cell lines BT20 (RRID: CVCL_0178, cat#HTB-19), MDA-MB-468 (RRID: CVCL_0419, cat#HTB-25), MDA-MB-175-VII (RRID: CVCL_1400, cat#HTB-132), and human choriocarcinoma cells, Bewo (RRID: CVCL_0044, cat#CCL-98), were obtained from ATCC and tested negative for mycoplasma contamination. PANC1 and PANC10.05 cells were grown in DMEM (Gibco, cat#11965-092) or RPMI (Gibco, cat#11875-093) media containing 10% heat inactivated BenchMark FBS (Gemini, cat#100-106). Bewo and BT20 cells were cultured in Ham’s F-12K (Gibco, cat#21-127-022) or EMEM (ATCC, cat#30-2003) media, respectively, also supplemented with 10% FBS. Retroviral-producing cells, Phoenix Eco (RRID: CVCL_H717, cat#CRL-3214) and PG-13 (RRID: CVCL_4273, cat#CRL-10686) cells were obtained from ATCC and tested negative for mycoplasma contamination. The cells were cultured in DMEM media supplemented with 10% FBS. Cell lines were authenticated by the Cytogenetic and Cell Authentication Core at MD Anderson Cancer Center. MycoAlert Mycoplasma Detection Kit (Lonza, cat#LT07-318) was used to test cells for mycoplasm throughout the study.

### Cloning of human VGLL1-Myc cDNA into retrovirus MG-neo vector

Human VGLL1 with MYC tag containing restriction enzyme sites Not1 and Sal1 (hVGLL1-MYC, sequence below) was synthesized by GeneArt and inserted in pENTR221 vector. NEB-10 beta competent cells (New England Biolabs, cat#C3019H) were used to expand the vectors following manufacturer protocol. To clone hVGLL1-MYC cDNA into retrovirus MG-neo vector, hVGLL1-MYC pENTR221 and MG-neo plasmids were digested using Not1-HF (New England Biolabs, cat#R3189S) and Sal1-HF (New England Biolabs, cat#R3138S) for 1 hour at room temperature. Gel purified hVGLL1-MYC was ligated to the MG-neo vector destination plasmid using T4 DNA ligase (New England Biolabs, cat#101228-180) for 1 hr at room temperature. Next, bacteria transformation was performed for hVGLL1-MYC MG-neo plasmid expansion, and the plasmid was purified using Wizard Plus SV Minipreps DNA purification systems (Promega, cat#A1470). To verify the hVGLL1-MYC insert sequence, the plasmid was digested using restriction enzymes Not1 and Sal1 and ran on a 1% agarose gel (Lonza, cat#50002) followed by purification by QIAquick Gel Extraction Kit (Qiagen, cat# 28704). The insert sequence was confirmed using Advanced Technology Genomics Core at MD Anderson Cancer Center using Forward primer: AATTCGCCAGCACAGTGGAGATC and Reverse primer: CGGCAATATGGTGGAAAATAACCGG. The sequencing results were viewed on Chromas 2.6.6 (RRID: SCR_000598) software and sequences were aligned using nucleotide BLAST (BLASTn suite, RRID: SCR_001598) (18). Next, hVGLL1-MYC MG-neo plasmid DNA was expanded and purified using a high-speed plasmid midi kit (Qiagen, cat#12643). The plasmid concentration was determined using a nanodrop.

### cDNA hVGLL1-MYC Sequence with restriction enzyme sites (bold):

TC**GCGGCCGC**CATGGAAGAAATGAAGAAGACTGCCATCCGGCTGCCCAAAGGCAAACAGAAGCCTATAAAGACGGAATGGAATTCCCGGTGTGTCCTTTTCACCTACTTCCAAGGGGACATCAGCAGCGTAGTGGATGAACACTTCTCCAGAGCTCTGAGCAATATCAAGAGCCCCCAGGAATTGACCCCCTCGAGTCAGAGTGAAGGTGTGATGCTGAAAAACGATGATAGCATGTCTCCAAATCAGTGGCGTTACTCGTCTCCATGGACAAAGCCACAACCAGAAGTACCTGTCACAAACCGTGCCGCCAACTGCAACTTGCATGTGCCTGGTCCCATGGCTGTGAATCAGTTCTCACCGTCCCTGGCTAGGAGGGCCTCTGTTCGGCCTGGGGAGCTGTGGCATTTCTCCTCCCTGGCGGGCACCAGCTCCTTAGAGCCTGGCTACTCTCATCCCTTCCCCGCTCGGCACCTGGTTCCAGAGCCCCAGCCTGATGGGAAACGTGAGCCTCTCCTAAGTCTCCTCCAGCAAGACAGATGCCTAGCCCGTCCTCAGGAATCTGCCGCCAGGGAGAATGGCAACCCTGGCCAGATAGCTGGAAGCACAGGGTTGCTCTTCAACCTGCCTCCCGGCTCAGTTCACTATAAGAAACTATATGTATCTCGTGGATCTGCCAGTACCAGCCTTCCAAATGAAACTCTTTCAGAGTTAGAGACACCTGGGAAATACTCACTTACACCACCAAACCACTGGGGCCACCCACATCGATACCTGCAGCATCTTGAG**GGATCC**GGAGGAGGCGGATCT**GAGCAGAAACTCATCAG** **TGAAGAGGACCTG**TGA**GTCGAC**G

Not1 (GCGGCCGC)

BamH1 (GGATCC)

MYC (GAGCAGAAACTCATCAGTGAAGAGGACCTG)

Sal1 (GTCGAC)

### Production of hVGLL1-MYC MG-neo retrovirus

For retrovirus production, Phoenix Eco cells plated in a 60 mm dish were incubated overnight to reach about 60-80% confluency. To transduce the cells, 5ug of hVGLL1-MYC MG-neo plasmid DNA and 30ul TransIT-293 (Mirus bio, Qiagen, cat#MIR2704) transfection reagent were each diluted in 300uL of Opti-MEM (Gibco, cat#51985-034) in separated tubes. After 10 minutes incubation at room temperature, the diluted DNA was added into a tube containing the diluted transfection reagent in a drop-wise fashion and incubated for another 10 minutes at room temperature. Next, media from Phoenix Eco cells was replaced with 1mL Opti-MEM and 600uL of the DNA mixture was added to the cells in a drop-wise fashion and cells were cultured overnight in the incubator. The next day, media were changed to DMEM with 10% FBS (without antibiotics) and the cells were incubated overnight. The next day, the supernatant from hVGLL1-MYC MG-neo transduced Phoenix Eco cells was collected, filter sterilized and mixed with 10ug/mL polybrene (Sigma, cat#TR-1003-G). To generate stock of viral-producing cell we transduced PG-13 cells with the supernatant prepared from transduced Phoenix Eco cells mixed with polybrene. Human VGLL1 gene in these PG-13 hVGLL1-MYC MG-neo cells was detectable after 48 hr.

### Transduction of PANC1 cells with hVGLL1-MYC MG-neo retrovirus plasmid

To generate PANC1 cells overexpressing hVGLL1-MYC for the experiments, overnight cultured Panc1 cells with 80% confluency were incubated with the filtered supernatant from PG-13 hVGLL1-MYC MG-neo cells mixed with polybrene. PANC1 cells expressing hVGLL1-MYC were selected by cell culture in the presence of G418 sulfate antibiotic at a concentration of 0.5mg/ml for 4 days.

### siRNA knockdown

PANC10.05, BT20, and Bewo cells were plated in 6-well plates and cultured overnight. The cells at 60-80% confluency were used for transfection with siVGLL1 (ThermoFisher Scientific, cat#4392420 s28152) or siScramble control (ThermoFisher Scientific, cat#4390846). Briefly, 9uL lipofectamine RNAiMAX transfection reagent (Invitrogen, cat#13778030) and 30pmol of siVGLL1 (ThermoFisher Scientific, cat#4392420 s28152) or siScramble (ThermoFisher Scientific, cat#4390846) were diluted in 150uL Opti-mem media (Gibco, cat#51985-034). Next, diluted siRNA was added to diluted lipofectamine RNAiMAX at 1:1 ratio and incubated for 5 minutes at room temperature. 250uL of combined siRNA and lipofectamine were added in a drop-wise fashion into the cells. The transfected cells incubated with the siRNA solution for 48 hrs were used for the indicated experiments.

### Western blot

The indicated cancer cells were plated on 6-well plates at a density to reach about 90% confluency after 48h cultured. Next, the cells were washed twice with cold PBS (Corning, cat#21-040-CV) and lysed with 350uL of lysis buffer (50mM HEPES (Corning, cat#25-060-C1), 150mM NaCl (Invitrogen, cat#AM9760G), 1mM EDTA (Sigma, cat#03690-100ml), 1% triton X100 (Millipore, cat#T9284-100ml), 1mM PMSF (Sigma, cat#P-7626), and 1X Halt protease and phosphatase inhibitor (ThermoScientific, cat#78-138) for 45 minutes on ice. Protein concentrations were quantified using BCA assay (ThermoFisher) with BSA standard set (Bio-Rad). 10ug of protein from each sample was loaded into well of 10% Tris-Glycine gel (Invitrogen) and ran at 125V. The resolved proteins were transferred to a PVDF membrane (BioRad, cat#1704156) for 7 minutes at 25V using Bio-Rad Trans-Blot Turbo Transfer System (cat#1704150). Next, the membrane was blocked with EveryBlot Blocking Buffer (Bio-Rad) for 30 min at room temperature and probed with primary antibodies against VGLL1 (ProteinTech, cat#10124-2AP, RRID: AB_2218174), MYC (Cell Signaling, cat#2276), or GAPDH (Cell Signaling, cat#5174, RRID: AB_10622025) overnight at 4°C followed by incubation with anti-mouse (Cell Signaling, cat#7076, RRID: AB_330924) or anti-rabbit (Cell Signaling, cat#7074, RRID: AB_2099233) HRP-conjugated antibodies for 45 min at room temperature. Immunoblots were developed using Clarity Western ECL Substrate (Biorad, cat#1705060) and imaged by ChemiDoc MP (Biorad, cat#12003154).

### VGLL1 immunoprecipitation

For immunoprecipitation (IP) of VGLL1 protein from cancer cells we used Pierce™ Classic Magnetic IP/Co-IP Kit (ThermoFisher Scientific, cat#88804). VGLL1 was IP from 200ug of beads precleared cell lysate using 5ug VGLL1 Ab (ProteinTech, cat#10124-2AP, RRID: AB_2218174) per sample. VGLL1-IP proteins were washed and eluted from the beads with Laemmli sample buffer. The resolved protein was detected by western blot with anti-VGLL1 Ab. We also included IP control samples without VGLL1 Ab to determine potential non-specific protein binding to the beads. Sample inputs contained 10ug of protein from the indicated cell lysate.

### Real-time quantitative PCR

Real time quantitative PCR (RT-qPCR) was performed to determine hVGLL1 mRNA expression in PANC1, PANC10.05, BT20, Bewo cells. RNA was isolated from the cells cultured on 6-well plates (80-90% confluency) using RNAeasy Plus mini kit (Qiagen, cat#74134) according to the manufacturer’s protocol. The sample RNA was qualified using a NanoDrop Onec (ThermoFisher). High-capacity RNA-to-cDNA kit (Applied Biosystems, cat#4387406) was used to generate cDNA from 0.5ug RNA following the manufacturer’s instructions. cDNA was quantified using a NanoDrop. RT-qPCR amplifications were done with PowerUP SYBR Green Master Mix (Applied Biosystems, cat#A25742) using 500nM primers (see **Table 1**) and 10ng cDNA per reaction in a total volume of 20ul in 96-well plate. The samples were run in triplicate on a QuantStudio3 System (Applied Biosystems). VGLL1 mRNA expression was normalized to the mRNA expression level of GAPDH in the same sample. The results were then calculated as a relative VGLL1 mRNA expression compared to empty vector or siScramble controls that were expressed as 1 value.

**Table 1:** Table of primer sequences used in qRT-PCR.

Primer Name	Primer Sequence
Human GAPDH Forward	ACA ACT TTG GTA TCG TGG AAG G
Human GAPDH Reverse	GCC ATC ACG CCA CAG TTT C
Human VGLL1 Forward	CCA AAG GCA AAC AGA AGC CTA
Human VGLL1 Reverse	CAT CCAC ACC TTC ACT CTG ACT C
Human B-actin Forward	GCG AGA AGA TGA CC AGA TC
Human B-actin Reverse	CCA GTG GTA CGG CCA GAG G

### Proliferation assay

The effect of VGLL1 expression on cancer cell growth was determined by daily counting of cells during seven days of culture. The cells were plated in triplicate at a density of 30,000 cells per well of 6-well plate. To count the cells, they were washed twice with PBS, trypsinized using 50uL of 0.25% Trypsin (Gibco, cat#325200-056), and collected with 1mL media. After centrifugation, the cell pellets were resuspended in 50uL of media and 50uL of 0.2% trypan blue (Lonza BioWhittaker, cat#17-942E). 20uL of the cell suspension was transferred into the cellometer slide (Nexcelom, cat#SD100) and analyzed for the number of live cells by a Nexelom cell counter.

### Invasion assay

CHEMICON cell invasion assay (Millipore Sigma cat#ECM550) was used to determine the effect of VGLL1 expression on cancer cell invasion. Briefly, 500,000 cells were plated in 300uL serum-free media on top of a transwell coated with a basement membrane matrix and the bottom chamber was filled with 500ul media supplemented with 10% FBS. After 24h incubation, migrated cells on the bottom of the transwell were stained with the cell staining solution provided in the kit. Images were acquired using an Axiovert 200 microscope and quantified using ImageJ software (RRID: SCR_003070). Samples were plated in triplicate and four representative images were taken from each well.

### Chromatin immunoprecipitation

For VGLL1 chromatin immunoprecipitation sequencing (ChIP-Seq) analysis, PANC10.05, BT20 and Bewo cells were sent to Active Motif (Carlsbad, CA). To prepare chromatin, the cells were fixed with 1% formaldehyde for 15 min and quenched with 0.125 M glycine. Chromatin was isolated by adding lysis buffer and disrupted with a Dounce homogenizer. Lysates were sonicated and the DNA sheared to an average length of 300-500 bp with Active Motif’s EpiShear probe sonicator (cat#53051). Genomic DNA (Input) was prepared by treating aliquots of chromatin with RNase, proteinase K, and heat for de-crosslinking, followed by SPRI beads clean up (Beckman Coulter) and quantitation by Clariostar (BMG Labtech). For ChIP, 30 ug chromatin was precleared with protein G agarose beads (Invitrogen) and genomic DNA regions of interest were isolated using 5ug of VGLL1 Ab (Protein Tech, cat#10124-2-AP). IP complexes were washed, eluted from the beads with SDS buffer, and subjected to RNase and proteinase K treatment. Crosslinks were reversed by incubation overnight at 65°C. DNA from ChIP was purified by phenol-chloroform extraction and ethanol precipitation.

Quantitative PCR (QPCR) reactions were carried out only for PANC10.05 in triplicate on specific genomic regions using SYBR Green Supermix (Bio-Rad). The resulting signals were normalized for primer efficiency by carrying out QPCR for each primer pair using Input DNA.

### ChIP sequencing (RRID: SCR_001237)

Illumina sequencing libraries were prepared from the ChIP and Input DNAs by the standard consecutive enzymatic steps of end-polishing, dA-addition, and adaptor ligation. Steps were performed on an automated system (Apollo 342, Wafergen Biosystems/Takara). After a final PCR amplification step, the resulting DNA libraries were quantified and sequenced on Illumina’s NovaSeq 6000 (75 nt reads, single end). Reads were aligned to the human genome (hg38) using the BWA algorithm (default settings, RRID: SCR_010910) (19). Duplicate reads were removed, and only uniquely mapped reads (mapping quality >= 25) were used for further analysis. Alignments were extended in silico at their 3’-ends to a length of 200 bp, which is the average genomic fragment length in the size-selected library and assigned to 32-nt bins along the genome. The resulting histograms (genomic “signal maps”) were stored in bigWig files. Peak locations were determined using the MACS algorithm (v2.1.0) (20) with a cutoff of p-value = 1e-7. Peaks that were on the ENCODE blacklist of known false ChIP-Seq peaks were removed. Signal maps and peak locations were used as input data to Active Motifs proprietary analysis program, which creates Excel tables containing detailed information on sample comparison, peak metrics, peak locations and gene annotations. Other key software used: bcl2fastq2 (v2.20) (processing of Illumina base-call data and demultiplexing), Samtools (v0.1.19, RRID: SCR_002105) (processing of BAM files), BEDtools (v2.25.0, RRID: SCR_006646) (processing of BED files), wigToBigWig (v4) (generation of bigWIG files).

ChIP-Atlas was used to compare our ChIP-seq data to publicly available ChIP-seq datasets using ChIP-Atlas online tool (https://chip-atlas.org/enrichment_analysis, RRID: SCR_015511) (21, 22).

### RNA sequencing

RNA sequencing analysis from total RNA isolated from different cancer cells was performed by Active Motif (Carlsbad, CA). For each sample, 500ng of total RNA was then used in Illumina’s TruSeq Stranded mRNA Library kit (Cat# 20020594). Libraries were sequenced on Illumina NovaSeq 6000 as paired-end 150-nt reads. Sequence reads were analyzed with the STAR alignment–DESeq2 software pipeline (RRID: SCR_004463). GO analysis was used to analyze several gene sets using the GO online tool (http://geneontology.org/).

### Statistical analyses

Graphpad Prism 10 (RRID: SCR_000306) was used to perform unpaired t test analysis for cell invasion assays and two-way ANOVA analysis for proliferation assays. A p<0.05 was considered statistically significant.

## Results

### VGLL1 expression promotes tumor cell proliferation and invasion

**Figure 1:**
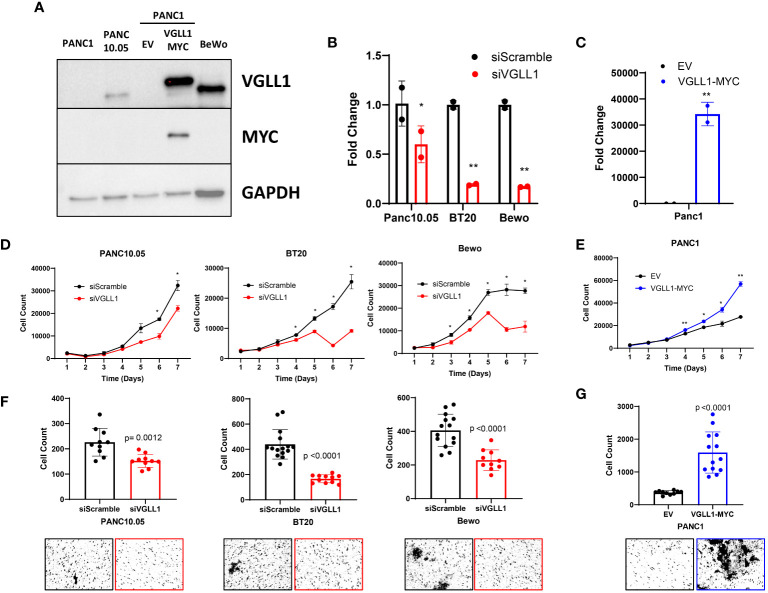
VGLL1 expression drives tumor cell proliferation and invasion. **(A)** Western blot analysis to assess VGLL1 protein expression in the cell lines PANC1, PANC10.05, Bewo, and PANC1 cells transduced to express either hVGLL1-MYC or empty vector (EV). **(B)** RT-qPCR analysis of VGLL1 mRNA expression in siScramble- (black) or siVGLL1- (red) treated cells after 48h. **(C)** RT-qPCR analysis of VGLL1 mRNA expression after retroviral transduction of PANC1 cells with hVGLL1-MYC MG-neo plasmid (blue) or empty MG-neo vector (black). **(D, E)** Cell proliferation analysis of siScramble- (black) or siVGLL1-treated PANC10.05, BT20, and Bewo cells (red), and PANC1 cells transduced with empty MG-neo vector (black) or hVGLL1-MYC MG-neo plasmid (blue). **(F, G)** Cell invasion assay results comparing siScramble (black) or siVGLL1-treated PANC10.05, BT20, and Bewo cells (red), and PANC1 cells transduced with empty MG-neo vector (black) or VGLL1-MYC MG-neo plasmid (blue). The indicated cells were plated onto the top of a transwell chamber and after 24 hr incubation, migrated invading cells were stained. Images were taken and quantified using ImageJ, with representative images displayed. *p<0.05, **p<0.01.

This study aimed to identify common and unique VGLL1-dependent functions in tumor cells derived from 3 different cancer types that may contribute to disease progression. Based on the elevated VGLL1 transcript expression reported in particular tumor types, we screened pancreatic cancer (PDAC), basal-like breast cancer (BLBC), and choriocarcinoma (a placenta-derived tumor) cells for VGLL1 protein expression. Western blot analysis demonstrated relatively high VGLL1 protein levels in PANC10.05 (PDAC), BT20 (BLBC), MDA-MB-468 (BLBC) and Bewo (choriocarcinoma) cells (**Supplementary Figure 1**). Based on these results we selected PANC10.05, BT20 and Bewo cells for our study, which represent cancer cells originating from pancreas, breast, and placenta tumors, respectively. By contrast, VGLL1 expression was undetectable in PANC1 PDAC cells (**Figure 1**; **Supplementary Figure 1**). Thus, PANC1 cells represent the ~60% of PDAC patients whose tumors do not express VGLL1. Since recent studies have suggested that VGLL1 is critical for the transition from classical to a more aggressive basal-like form of PDAC (11), we utilized PANC1 cells to assess phenotypic changes induced by ectopic VGLL1 overexpression.

Uncontrolled tumor cell proliferation and invasion are amongst the most important hallmarks of cancer progression. To determine how VGLL1 may impact these cellular processes and drive associated transcriptional changes, we utilized two *in vitro* models: siRNA knockdown and retrovirus overexpression systems. For downregulating VGLL1 expression, PANC10.05, BT20, and Bewo cells (expressing high levels of endogenous VGLL1) were transfected with the VGLL1-targeting siRNA or siScramble control. For overexpressing VGLL1, PANC1 cells were transduced with the hVGLL1-MYC MG-neo retroviral vector. RT-qPCR analysis of RNA isolated from these cells showed about 50% (PANC10.05) to 75% (BT20 and Bewo) reduction of VGLL1 transcript levels in cells transfected with siVGLL1 compared to siScramble control (**Figure 1B**). On the other hand, PANC1 cells transduced with the hVGLL1-MYC retroviral vector demonstrated more than a 3-log increase in VGLL1 mRNA levels compared to empty vector-transduced cells (**Figure 1C**). Furthermore, immunoblotting with anti-VGLL1 or anti-MYC Abs confirmed high VGLL1-MYC protein expression in transduced PANC1 cells (**Figure 1A**).

To evaluate the impact of VGLL1 expression on cancer cell proliferation, we monitored cell growth of these genetically altered and control cells for 7 days (**Figures 1D, E**). Tumor cells were plated at a density of 30,000 cells per well in a 6-well plate, and the number of live cells was quantitated daily. A 1.5-fold reduction in proliferation rate was observed by day 5 in BT20 and Bewo cells treated with siVGLL1 compared with control siScramble-treated cells (**Figure 1D**). A similar 1.7-fold reduction in proliferation of siVGLL1-treated PANC10.05 cells was observed by day 6 of culture (**Figure 1D**). Of note, in addition to inhibition of cell growth, a significant increase in cell death was observed in BT20 and Bewo cells by day 6 of culture leading to about 3-fold reduction in the number of live cells compared to siScramble-treated cells. This observation was consistent with our initial attempts at CRISPR-mediated complete VGLL1 knockdown, which was lethal to the cells (data not shown). Therefore, to maintain the integrity of tumor cell lines, all further experiments were performed within 48 hrs of siRNA knockdown. By contrast, ectopic overexpression of VGLL1 in PANC1 cells clearly increased the rate of tumor cell proliferation, with a greater than 2-fold increase in cell number by day 7 of culture compared to empty vector-transduced cells (**Figure 1E**). These results demonstrated that while augmentation of VGLL1 expression enhanced cell proliferation rate, downregulation of VGLL1 expression was associated with inhibition of cancer cell growth and induction of cell death.

Tumor metastasis is the primary cause of mortality in most cancer patients. Since cellular invasion is critical for the metastatic process (23), we next assessed the impact of modulating VGLL1 expression on this process. The invasion capacity of cells was measured by their ability to break down a basement membrane matrix and subsequently migrate through the pores on the bottom side of a transwell. Quantitative analysis of the cell invasion assay revealed that siVGLL1-treated PANC10.05, BT20, and Bewo cells all demonstrated a 1.5 to 2.6-fold decrease in invasion compared to control siScramble-treated cells (**Figure 1F**). By contrast, VGLL1-transduced PANC1 cells showed a 4.3-fold increase in migrated cells compared to empty vector-transduced control cells (**Figure 1G**). These results support the notion that VGLL1 overexpression in tumors may play a substantial role in promoting tissue invasion, consistent with its association with tumor aggressiveness and poor patient prognosis.

### VGLL1 interacts with distinct transcription factors to regulate transcription in different cancer cells

**Figure 2:**
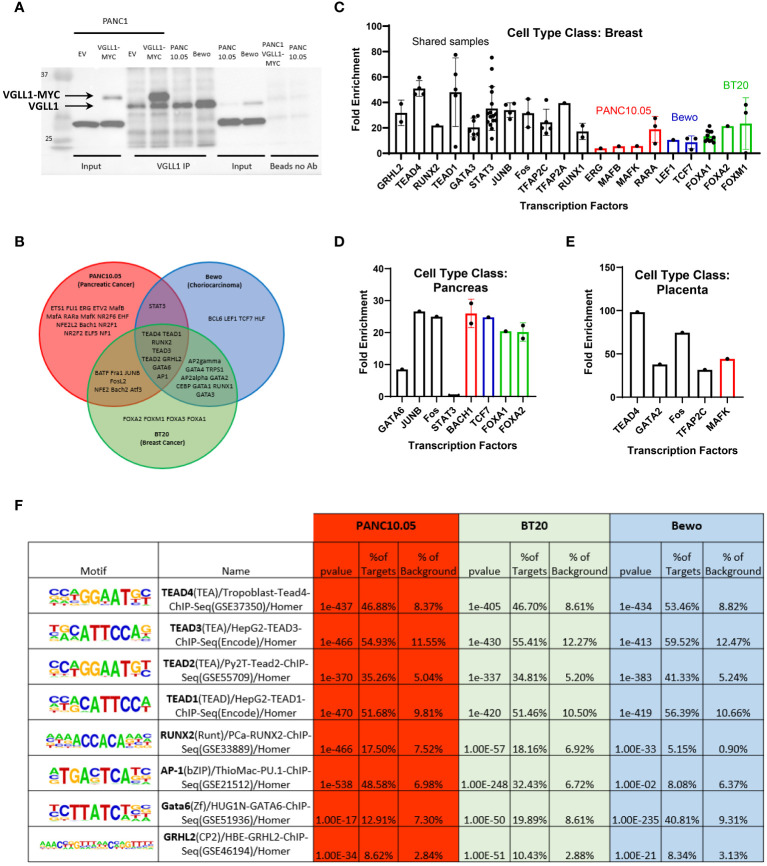
VGLL1 interacts with common and distinct transcription factors to regulate transcription in tumor cells. **(A)** Western blot analysis showing relative VGLL1 protein expression either in cell lysates or immunoprecipitated from PANC10.05 and Bewo cells expressing endogenous VGLL1, or PANC1 cells transduced with empty vector (EV) or hVGLL1-MYC plasmid (VGLL1-MYC). Samples without VGLL1 Ab (Beads no Ab) confirmed the specificity of the anti-VGLL1 Ab for IP applications. **(B)** VGLL1 ChIP-seq analysis was performed on native PANC10.05, BT20, and Bewo tumor cell lines. Homor Motif analysis of the VGLL1 chromatin binding regions revealed several transcription factors (TFs) likely to interact with VGLL1. The Venn diagram shows the common VGLL1 TFs between PANC10.05 (red), BT20 (green) and Bewo (blue) tumor cells. **(C–E)** ChIP-Atlas was used to compare our results with previously published ChIP-seq data. Each dot represents a different sample (cell line, tissue, etc.) used as a source to pull down individual TFs. Higher enrichment scores indicate higher similarity to the VGLL1 ChIP-seq results. Some samples demonstrated overlap in more than one tumor cell type (black) and others overlapped only with individual tumor cell lines PANC10.05 (red), BT20 (green), Bewo (blue). **(F)** Table of TFs that were identified in all three tumor cell types. The table contains the TF name and analyzed sample tissue, chromatin motif sequence recognized, percentage of overlapping targets compared to background and p-value. The same color scheme as above indicates the different tumor cell lines analyzed.

To identify the transcription factors (TFs) that can interact with VGLL1 in different cancer types, the chromosomal loci bound by this transcriptional co-activator were determined by chromatin immunoprecipitation sequencing (ChIP-seq) analysis performed on PANC10.05, BT20, and Bewo tumor cells. First, we validated the utility of anti-VGLL1 Ab by evaluating its specificity by Western blot analysis. As shown in **Figure 2A**, VGLL1 immunoprecipitation from lysates of both PANC10.05 and Bewo cells showed a single predominant band representing the native form of VGLL1 protein at approximately 29 kDa, consistent with its predicted molecular weight. As expected, MYC-tagged VGLL1 protein derived from transduced PANC1 cells migrated slower at about 32 kDa. A nonspecific band was observed at approximately 26 kDa in all input cell lysates; however, no such band was detected in the VGLL1 immunoprecipitated samples (**Figure 2A**). Further validation of the VGLL1 Ab was performed by rapid immunoprecipitation mass spectrometry of endogenous proteins (RIME) on VGLL1-MYC transduced PANC1 cells. The mass spectrometry results demonstrated a 68% coverage of the VGLL1 protein, including 28 unique peptides (data not shown). Collectively, these results confirmed that the VGLL1-specific antibody met the quality standards required for ChIP-seq analysis.

HOMER motif analysis performed on the ChIP-seq data derived from PANC10.05, BT20 and Bewo cells revealed multiple potential binding partners for VGLL1. The Venn diagram in **Figure 2B** depicts all the TFs that showed highly significant p-values of less than 1E-10. Some TFs were specific for individual cell lines, others were shared between two cell lines, and a total of eight TFs were shared amongst all three tumor types, including TEAD1, TEAD2, TEAD3, TEAD4, GRHL2, RUNX2, AP1, and GATA6. To validate our findings, we used ChIP-Atlas Enrichment analysis to compare our data to publicly available ChIP-seq data (21, 22). This analysis compares prior TF immunoprecipitation results derived from different tissue types or cell lines to our findings, with higher fold enrichment scores indicating a higher degree of similarity to our VGLL1 ChIP-seq data (**Figures 2C–E**). The breast-derived samples showed a high degree of similarity for multiple TFs, as shown in **Figure 2C**. Each dot represented a single database entry for the indicated specific TF, with results distinguished by TFs found to bind VGLL1 in multiple tumor cell lines (black), or in individual cell lines: PANC10.05 (red), BT20 (green) and Bewo (blue). High ChIP-Atlas enrichment scores were also observed for multiple TFs associated with pancreas and placenta, though the database entries for these tissue types were significantly less (**Figures 2D, E**). Overall, a significant degree of overlap was observed in the TF profiles from the three tumor cell lines found in our HOMER motif analysis and those identified in tissue-specific ChIP-seq database. By contrast, low enrichment scores were seen in the breast tissue-derived ChIP-seq database samples for TFs found only in the PANC10.05 cell line (for example, MAFB and ERG, **Figure 2C**).

Comparing the eight TFs identified to interact with VGLL1 in all three tumor cell lines, we assessed the similarity in target loci found in the different cancer cells with publicly available ChIP-seq data for the same TFs (**Figure 2F**). This comparison showed a very high degree of overlap in the percentage of target loci where VGLL1 bound compared to those targets previously identified for TEAD1, TEAD2, TEAD3, and TEAD4 (*p* < 10e-370 for all 3 tumor cell lines). For example, the VGLL1 ChIP-seq analysis of PANC10.05 cells demonstrated a 46.88% overlap with a TEAD4 ChIP-seq analysis from placental trophoblast cells and low background binding outside these targets. Similar results were observed for BT20 and Bewo cells (46.70% and 53.46% target overlap with TEAD4, respectively). The high degree of overlap observed for the 4 TEADs in all 3 tumor cell lines suggest that VGLL1 interacts with these TEADs globally and not in a tissue-specific manner (**Figure 2F**). Interestingly, AP-1 targets identified from ChIP-seq overlapped significantly with VGLL1 target loci in PANC10.05 and BT20 cells (48.58% and 32.43%, respectively), but not Bewo cells. By contrast, GATA6 targets showed strong overlap with VGLL1 target loci identified in Bewo cells (40.81%), but less overlap in PANC10.05 cells and BT20 cells (12.91% and 19.89%, respectively). The TFs GATA3 and AP2gamma (TFAP2C) also demonstrated significant overlap with VGLL1 target loci in BT20 and Bewo cells, but not in PANC10.05 cells (**Figure 2F**). These results confirm previous studies reporting that VGLL1 can cooperate with TEAD4, GATA3 and TFAP2C to initiate transcription (7, 12, 24), and expand the list of candidate TFs that can potentially interact with the VGLL1 co-activator in either a global or tissue-specific fashion.

### VGLL1 regulates transcription of common genes in cancer cells derived from different tumor types

**Figure 3:**
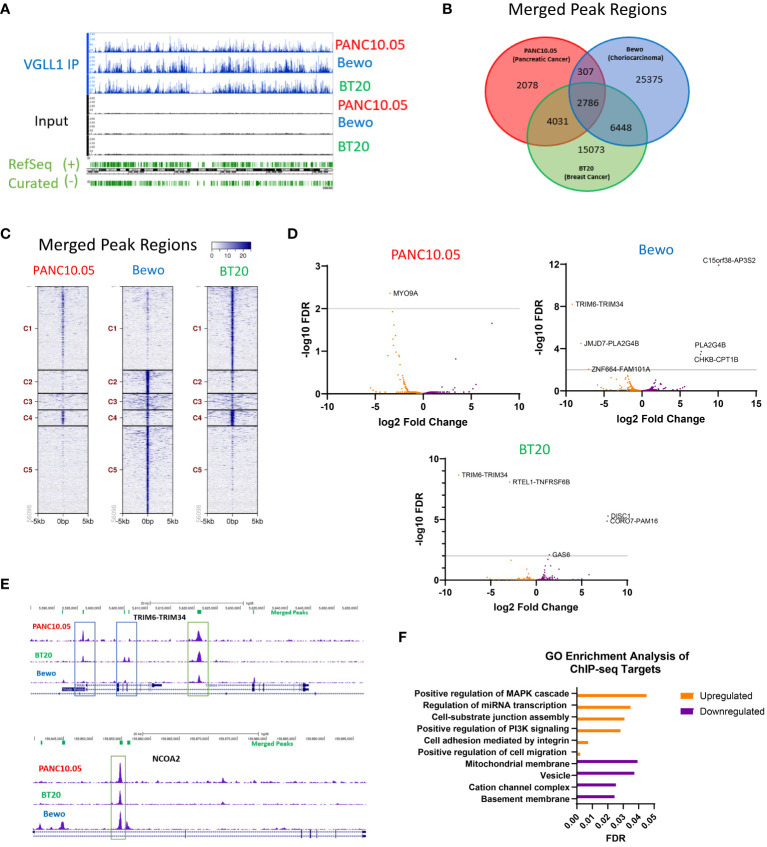
VGLL1 regulates transcription of common and unique genes in different tumor cell types. **(A)** A representative image of the ChIP-seq chromatin peaks detected for all tumor line samples comparing immunoprecipitated VGLL1 (VGLL1 IP) to input cell lysate. **(B)** Venn diagram shows the number of merged peaks detected and overlap between the 3 cell lines PANC10.05 (red), BT20 (green) and Bewo (blue). **(C)** Comparison of merged peak regions showing common and unique VGLL1 binding clusters for each tumor cell line. **(D)** RNA-seq analysis was performed on the indicated tumor cell lines treated with either siVGLL1 or siScramble. Changes in transcript expression for genes associated with VGLL1-binding regions identified in the merged peaks analysis are shown as Volcano plots for each tumor cell line, illustrating genes upregulated (orange) or downregulated (purple) by VGLL1 expression. **(E)** Representative images of the ChIP-seq chromatin peaks for each tumor cell line are shown for TRIM6-TRIM34, a read-through transcript upregulated by VGLL1 expression (top), NCOA2, a gene whose expression was upregulated by VGLL1 expression (bottom). Green boxes highlight examples of merged peaks present in all 3 tumor cell types and blue boxes indicate peaks not present in all cell lines. **(F)** GO-enrichment analysis was performed on VGLL1 ChIP-seq target genes modulated in response to VGLL1 knockdown. Upregulated pathways are shown in orange and downregulated pathways are in purple. .

We next analyzed the ChIP-seq data from PANC10.05, BT20 and Bewo cells to explore the exact location of the VGLL1-TF complex binding to specific genes. **Figure 3A** shows a representative image of the ChIP-seq peaks showing the specificity of immunoprecipitated VGLL1 compared with input control cell lysates from the 3 tumor cell lines. Notably, the number of chromatin binding regions identified corresponded to the level of VGLL1 expression (**Figure 3B**); BT20 and Bewo cells, demonstrating the highest VGLL1 expression, showed approximately 28,000 and 35,0000 total merge peaks, respectively compared to ~9,000 total merged peaks for PANC10.05 cells, which express lower levels of VGLL1 (**Figure 3B**). Importantly, VGLL1 bound at 2786 specific gene loci that were common to all three cell lines (**Figure 3B**). **Supplementary Table 1** contains a comprehensive list of genes shown to bind VGLL1, as determined by ChIP-seq analysis (**Supplementary Table 1**). Unexpectedly, most of the merged peak regions were found within the intron regions of genes rather than annotated promoter regions (**Supplementary Figures 2A-C**). Co-transcriptional activators such as YAP1 typically show a higher proportion of interactions at promoter regions than introns (25); however, VGLL1 interacted mainly with introns and distal intergenic regions in all 3 cell types studied (**Supplementary Figure 2A**). Peak-centered histograms of VGLL1 ChIP-seq peaks revealed significant overlap between all cell lines with no enrichment observed in the input samples (**Supplementary Figure 2C**). These results also demonstrated a significant increase in merged peak intron regions over the number of promoter region interactions (**Supplementary Figure 2C**). A ChIP-seq peaks heatmap of gene regions using coordinates centered around VGLL1 +/-5Kb were organized into five clusters and sorted by descending average value within each cluster for each of the 3 tumor cell lines (**Figure 3C**; **Supplementary Figure 2B**). Again, the promoter region heatmap demonstrated relatively few VGLL1 interactions (**Supplementary Figure 2B**). However, the merged region heatmap revealed tissue-specific clustering; as shown in **Figure 3C**, Cluster 4 corresponded to the overlapping binding regions found within all 3 cell lines. By contrast, Cluster 1 corresponded to BT20-specific binding regions and Cluster 5 was enriched in Bewo-specific binding regions.

To identify transcriptional regulation events potentially impacted by VGLL1, we compared the VGLL1 ChIP-seq findings to RNA-seq data obtained from PANC10.05, BT20, and Bewo cells treated with siVGLL1 versus siScramble. Volcano plots showing differential gene transcript expression of VGLL1-bound genes identified by ChIP-seq demonstrated that many of the genes were regulated in a similar manner. For example, the TRIM6-TRIM34 fusion transcript was upregulated by VGLL1 in both BT20 and Bewo cells. (**Figure 3D**). Since VGLL1 expression levels impacted the number of chromatin binding locations identified, separate plots included VGLL1-binding regions identified in two of the three tumor cell lines (**Supplementary Figure 2D**). For each of the 3 cell lines, genes downregulated or upregulated by siVGLL1-knockdown are indicated (**Supplementary Figure 2D**). **Supplementary Table 2** contains the complete list of genes impacted by modulation of VGLL1 expression, and a brief description of protein function. **Figure 3E** shows representative VGLL1 ChIP-seq signal peak tracks for two genes, TRIM6-TRIM34 and NCOA2, that were both shown to bind VGLL1 in all three tumor cell lines. Interestingly, while TRIM6-TRIM34 transcript levels were upregulated in BT20 and Bewo cells, NCOA2 expression was upregulated in PANC10.05 and Bewo cell lines (**Figure 3E**). Furthermore, some of the observed intragenic VGLL1-binding peaks were present in all 3 cell lines, while others were detected in only two of the three lines (**Figure 3E**).

To identify pathways directly regulated by VGLL1 expression, gene ontology (GO) analysis was performed on genes that were identified by ChIP-seq analysis to be bound by VGLL1 and that were either upregulated or downregulated at the transcript level following VGLL1 knockdown in at least two of the three tumor cell lines. This analysis revealed a potential role for VGLL1 in upregulating genes involved in cell migration, MAPK and PI3K signaling pathways (**Figure 3F**). On the other hand, genes involved in basement membrane and cation channel complex function were downregulated by VGLL1 expression. These findings were consistent with our functional results demonstrating that VGLL1 expression enhanced tumor cell invasion capacity (**Figures 1F, G**).

### VGLL1 expression regulates transcription of genes involved in cell proliferation and invasion

**Figure 4:**
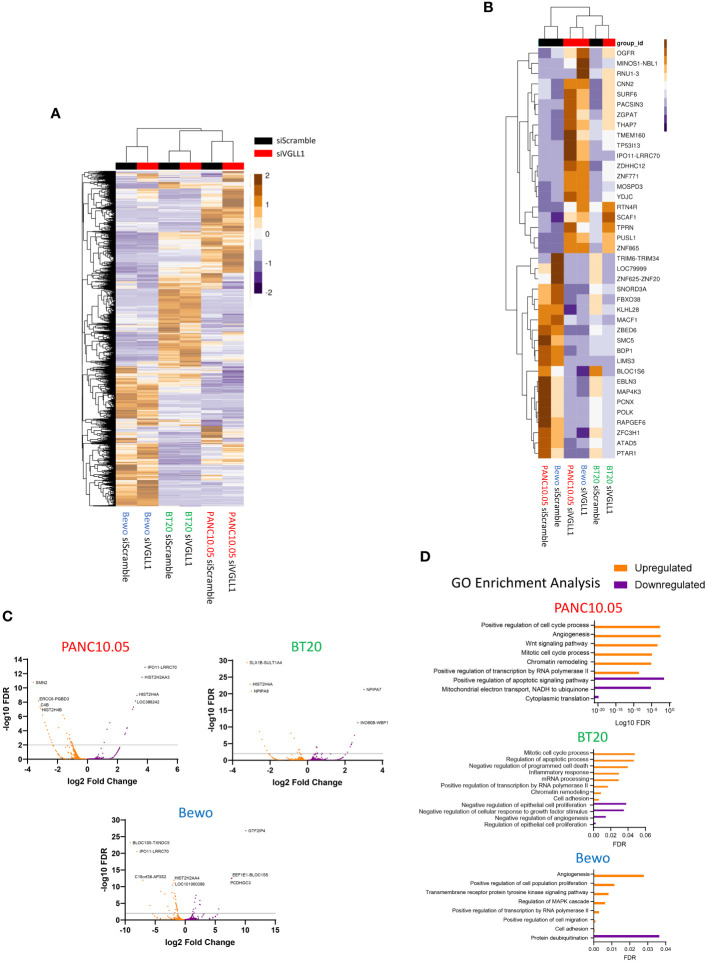
VGLL1 expression regulates transcription of genes involved in cellular proliferation and invasion. **(A)** Global heatmap showing all differentially expressed genes (DEGs) identified from RNAseq analysis of PANC10.05, BT20, and Bewo cells following treatment with either siScramble or siVGLL1. **(B)** Heatmap showing the top 20 upregulated or downregulated genes in response to VGLL1 knockdown that were common to all three tumor cell lines analyzed. **(C)** Volcano plots of DEGs identified in each tumor cell line. **(D)** Results of GO-pathway analysis using the DEGs identified for each tumor cell line. Upregulated genes and pathways are shown in orange and downregulated genes and pathways are indicated in purple.

To determine the overall impact of VGLL1 expression on the transcriptomic profiles of the different cancer cells, global RNA-seq data from PANC10.05, BT20, and Bewo cells treated with siScramble versus siVGLL1 was analyzed without prior filtering on the genes shown to bind VGLL1 by ChIP-seq. The results for all differentially expressed genes (DEGs) for each cell line are shown in **Figure 4A**. This analysis revealed several gene clusters that were either upregulated or downregulated by VGLL1 in a global or tumor cell-specific fashion. The top 20 overall DEGs for each tumor cell line are depicted in **Figure 4B**. In addition, volcano plots for each cell line in **Figure 4C** illustrate the upregulation or downregulation of specific gene transcription in response to VGLL1 expression.

To better understand these VGLL1-dependent genes, we sorted the list of DEGs by protein function, highlighting the genes that were also identified by ChIP-seq analysis to interact with VGLL1 (**Supplementary Table 2**). While 34 of the 128 genes (26.6%) were either uncharacterized or were predicted not to form a protein, 39 of the genes (30.5%) were known to be involved with transcription or other nuclear processes. Interestingly, 28 of the genes (21.9%) had known roles in cell proliferation and invasion, while 13 genes (10.2%) were involved with developmental processes. Moreover, gene ontology (GO) analysis performed on DEGS within each tumor cell line revealed a number of pathways upregulated in multiple cell lines, including angiogenesis, chromatin remodeling, positive regulation of transcription, in addition to cell proliferation and invasion. (**Figure 4D**). Furthermore, several of the genes identified as being potentially regulated by VGLL1 were not included in the GO analyses, most notably TRIM6-TRIM34, NCOA2, and ASLX2. These genes were not only highly upregulated by VGLL1 at the mRNA level but were also identified in the ChIP-seq analysis, indicating that VGLL1-TF complexes can interact with them directly. Interestingly, several studies have shown these genes to have roles in cancer cell proliferation, invasion, angiogenesis, and EMT transition (26–38). Taken together, our findings provide additional mechanistic evidence that VGLL1 plays an important role in modulating the expression of specific genes involved in driving tumor progression.

## Discussion

Transcriptomic analyses of normal human tissues have shown that the co-transcriptional activator VGLL1 demonstrates uniquely elevated expression within the placenta (4, 5, 39). VGLL1 is also overexpressed in multiple cancer types, demonstrating predominant expression in basal-like breast and pancreatic cancers with transcription increased by 8- to 60-fold compared to normal breast and pancreatic tissue (3, 4, 40). Emerging evidence has suggested that tumor cells may co-opt attributes of normal placental VGLL1 function to promote invasion, proliferation, and tumor progression (3, 8, 9). In this study, we demonstrated that modulation of VGLL1 expression has a marked impact on cell invasion and proliferation in multiple cancer cell types, and that it can interact with several known TFs to drive the expression of multiple genes involved in these pathways to promote tumor growth and metastasis (41).

Under normal physiological circumstances, VGLL1 acts as a master regulator of early placenta development. Yang et al. have demonstrated that VGLL1 plays an important role in placenta formation at the trophectoderm stage (7). Specifically, VGLL1 was shown to be critical for appropriate syncytiotrophoblast (SCT) and extravillous trophoblast (EVT) development since knockdown of VGLL1 during SCT and EVT differentiation led to a decrease in cell proliferation, failure to differentiate and widespread cell death (7). This was reminiscent of the reduced proliferation and cell death we observed in tumor cells following VGLL1 knockdown. Disruption of VGLL1 expression in the placenta can also lead to pathological conditions associated with preeclampsia and choriocarcinoma (42, 43). Choriocarcinoma is a trophoblastic malignancy that shows uncontrolled invasion and highly proliferative features unlike normal trophoblast cells, which demonstrate a controlled invasive phenotype during embryo implantation (44). In this study, we showed that VGLL1 is highly expressed in a choriocarcinoma cell line and that VGLL1 expression drove enhanced cell proliferation and invasion in these tumor cells. Further work will be required to characterize the precise mechanisms by which VGLL1 acts to promote these processes in both normal placental development and in placenta-derived tumors.

In addition to choriocarcinoma, VGLL1 has been shown to be highly overexpressed in a variety of other cancer types. Echoing its role in the placenta, VGLL1 expression has previously been linked to increased cell proliferation in basal-like breast cancer, estrogen receptor-positive breast cancer, gastric cancer, pancreatic cancer, prostate cancer and HPV-related cervical cancer (8–12, 24). In addition, VGLL1 overexpression has been shown to increase cell invasion in gastric cancer cells (9). However, the molecular mechanisms leading to these phenotypic changes have not been well-defined. Although its interactions with TEAD4 strongly suggest a primary function for VGLL1 as a co-transcriptional activator, its downstream target genes and potential interactions with other transcription factors are still largely unknown. Using a combination of cellular assays, ChIP-seq, and RNA-seq analyses, our study confirmed and extended prior findings by comprehensively assessing the downstream impact of VGLL1 expression in pancreatic, basal-like breast and placenta-derived cancer cells. We showed in all 3 tumor cell lines that downregulation of VGLL1 expression inhibited cell proliferation and invasion capacity. In addition, this study revealed that reduced VGLL1 expression led to a decrease in the transcription of several genes associated with cell growth, invasion, proliferation, and angiogenesis. Interestingly, many of the genes regulated by VGLL1 have not yet been mapped to a specific pathway but may play a substantial role in driving cancer progression.

TRIM6-TRIM34, a read-through transcript highly upregulated in the context of VGLL1 expression, encodes a fusion protein with no currently known function. However, TRIM6 and TRIM34 individually encode ubiquitin ligases that may play roles in cancer and placenta development. TRIM6 has been reported to increase cell proliferation, invasion, metastasis, and angiogenesis in cancer (26–30). In addition, it has been linked to maintaining pluripotent embryonic stem cells in mice (45). TRIM34 has been shown to facilitate cell fusion of epithelial cells, also known as multinucleated goblet cells (46). Interestingly, multinucleated trophoblast cells are required for embryo implantation and to develop a fused extravillous trophoblastic cell shell (47). The placenta also contains syncytiotrophoblast cells made up of fused cytotrophoblasts that are responsible for the transport of resources between the mother and fetus (48). Although it is currently unknown if TRIM34 plays a role in placental cell fusion, this gene does show moderate expression within normal human placenta (5). The ASXL2 gene, also shown to be upregulated by VGLL1, is reported to regulate EMT transition during trophoblast differentiation and has been linked to the promotion of tumorigenesis and cell proliferation in a variety of cancer types (31–34). This evidence collectively supports the notion that tumor cells may indeed co-opt VGLL1 function to promote invasion, proliferation, and tumor progression; however, further investigations into the roles and contributions of individual genes are required before firm conclusions can be made in this regard.

In addition to identifying individual candidate genes under the transcriptional control of VGLL1, ChIP-seq analysis identified chromatin targets throughout the genome capable of specifically interacting with VGLL1. Motif analysis of these interacting chromatin regions led to the identification of both known and novel TFs that may cooperate with VGLL1 to initiate transcription. For example, GATA3 and AP2 gamma (TFAP2C), two TFs that were recently reported to cooperate with TEAD4 and VGLL1 to differentiate pluripotent stem cells into trophoblast stem cells (7), were also identified from our ChIP-seq analysis of breast and placental tumor cells. Furthermore, previous studies have shown that VGLL1 can directly interact with TEAD1 and TEAD4 in cancer cells (7, 10, 12, 24). Our results validated these findings and expanded the list of TFs that VGLL1 can potentially interact with to include TEAD2, TEAD3, RUNX2, GATA6, AP-1 and GRHL2. These TFs have been linked to the hippo pathway and epithelial tissue development, PI3K/AKT pathway activation, and the promotion of tumorigenesis (49–51). Notably, we found that VGLL1 bound mostly at intron regions. Enhancer regions are enriched within intron regions, especially in tissue-specific gene regulation (52–55). Enhancers are clusters of transcription factor binding sites that activate the expression of target genes (52, 53, 56). This activation can impact RNA splicing, stabilize lncRNAs, and increase the rate of transcription (53, 54, 56–58). Further studies will be required to delineate how VGLL1 cooperates with these TFs at intron regions to drive normal development in the placenta and disease progression in cancer cells.

Clinical studies have shown that higher tumor VGLL1 expression correlates with lower overall survival in pancreatic cancer, gastric cancer, basal-like breast cancer, and breast cancers that become resistant to SERD therapy (4, 8, 9, 11, 24). Furthermore, our study demonstrated the dependency of tumor cells on VGLL1 expression by showing that downregulation of VGLL1 diminished cell invasiveness, proliferation, and survival. Together, these observations provide a compelling rationale for developing a VGLL1-targeted therapy for those cancer patients whose tumors demonstrate high VGLL1 expression. Attempts have been made to develop therapeutics against other co-transcriptional activators associated with cancer development, such as WWTR1 and YAP1 (59, 60). However, the high expression of these genes in most normal human tissues can induce toxicity, limiting the utility of such approaches. By contrast, VGLL1 demonstrates very low to negligible expression in most normal tissues apart from placenta, suggesting that targeting VGLL1 in tumors may prove feasible without causing dose-limiting side effects. Although many questions remain regarding the precise roles VGLL1 plays in promoting gene transcription, emerging evidence supports that effective targeting of this co-transcriptional activator may greatly benefit many patients suffering from different types of cancer.

## Data availability statement

The data that support the findings of this study are openly available in the Gene Expression Omnibus (GEO) repository at https://www.ncbi.nlm.nih.gov/geo/, reference numbers GSE267817 (ChIP-seq) and GSE267818 (RNA-seq).

## Author contributions

HS: Conceptualization, Data curation, Formal analysis, Funding acquisition, Investigation, Methodology, Validation, Visualization, Writing – original draft, Writing – review & editing. BP: Conceptualization, Data curation, Formal analysis, Investigation, Methodology, Supervision, Validation, Visualization, Writing – review & editing. BN: Data curation, Investigation, Methodology, Validation, Visualization, Writing – review & editing. YS: Data curation, Investigation, Methodology, Writing – review & editing. LE: Data curation, Formal analysis, Investigation, Methodology, Visualization, Writing – review & editing. AT: Investigation, Methodology, Writing – review & editing. AK: Project administration, Writing – review & editing. KB: Conceptualization, Methodology, Supervision, Writing – review & editing. GL: Conceptualization, Funding acquisition, Supervision, Writing – review & editing.
