# Supplementary material for: Placental co-transcriptional activator Vestigial-like 1 (VGLL1) drives tumorigenesis via increasing transcription of proliferation and invasion genes
Source: Front Oncol. 2024 Jun 7;14:1403052. doi: 10.3389/fonc.2024.1403052 (PMC11190739; doi:10.3389/fonc.2024.1403052)
Supplement: Supplementary file 2 [file Presentation_1.pptx]

## Slide 1
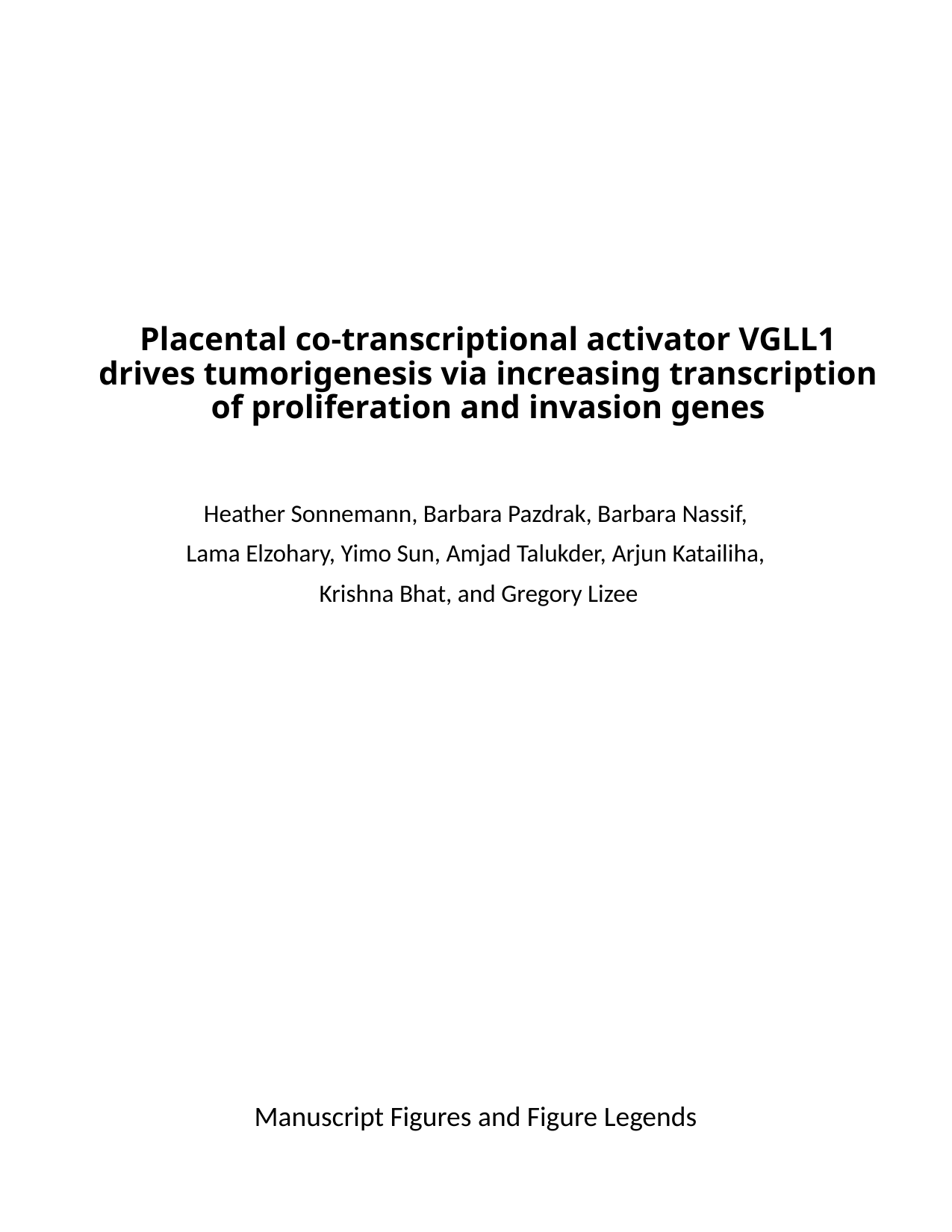

# Placental co-transcriptional activator VGLL1 drives tumorigenesis via increasing transcription of proliferation and invasion genes
Heather Sonnemann, Barbara Pazdrak, Barbara Nassif,
 Lama Elzohary, Yimo Sun, Amjad Talukder, Arjun Katailiha,
 Krishna Bhat, and Gregory Lizee
Manuscript Figures and Figure Legends

## Slide 2
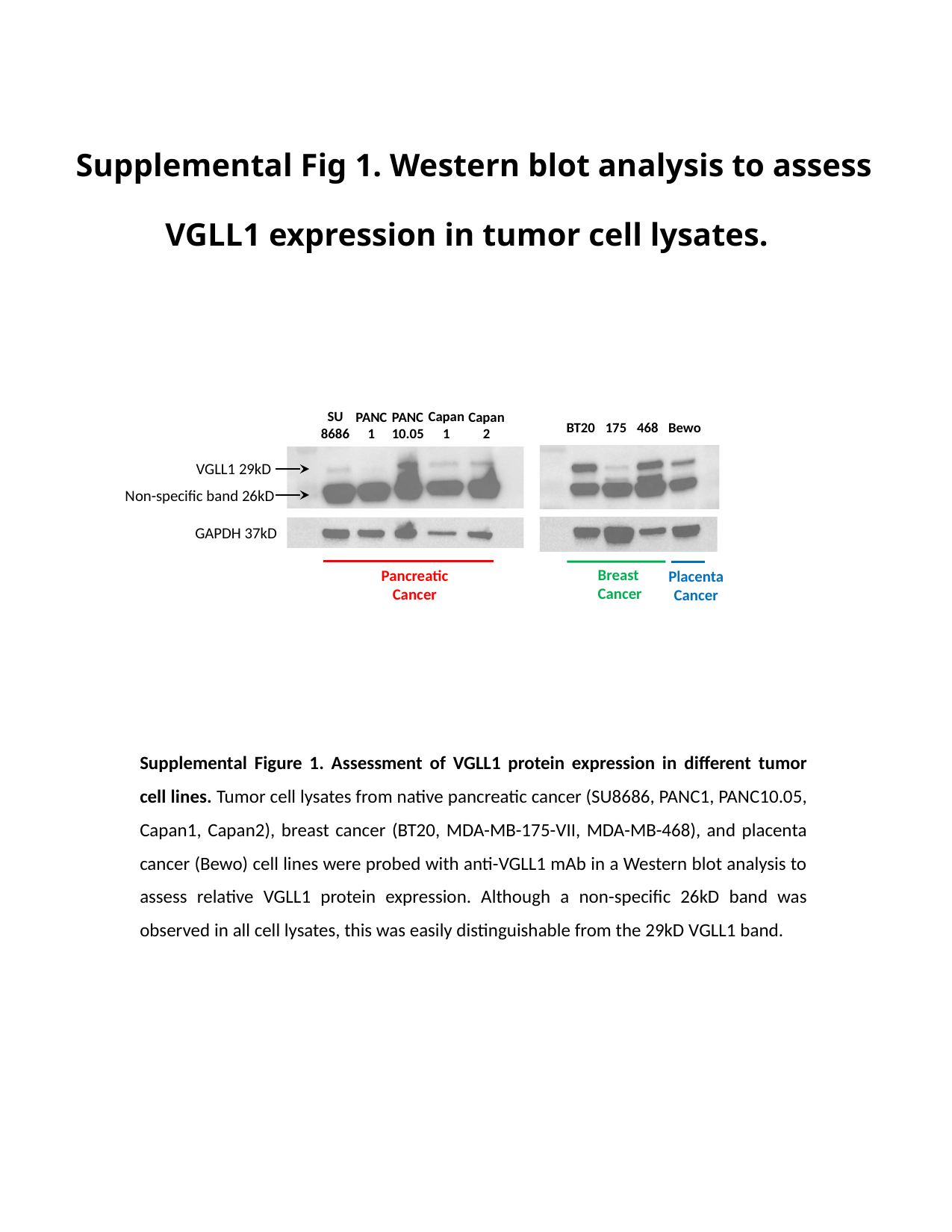

# Supplemental Fig 1. Western blot analysis to assess VGLL1 expression in tumor cell lysates.
SU
8686
Capan
1
PANC
1
Capan
2
PANC
10.05
BT20
175
468
Bewo
VGLL1 29kD
Non-specific band 26kD
GAPDH 37kD
Breast
Cancer
Pancreatic
Cancer
Placenta
Cancer
Supplemental Figure 1. Assessment of VGLL1 protein expression in different tumor cell lines. Tumor cell lysates from native pancreatic cancer (SU8686, PANC1, PANC10.05, Capan1, Capan2), breast cancer (BT20, MDA-MB-175-VII, MDA-MB-468), and placenta cancer (Bewo) cell lines were probed with anti-VGLL1 mAb in a Western blot analysis to assess relative VGLL1 protein expression. Although a non-specific 26kD band was observed in all cell lysates, this was easily distinguishable from the 29kD VGLL1 band.

## Slide 3
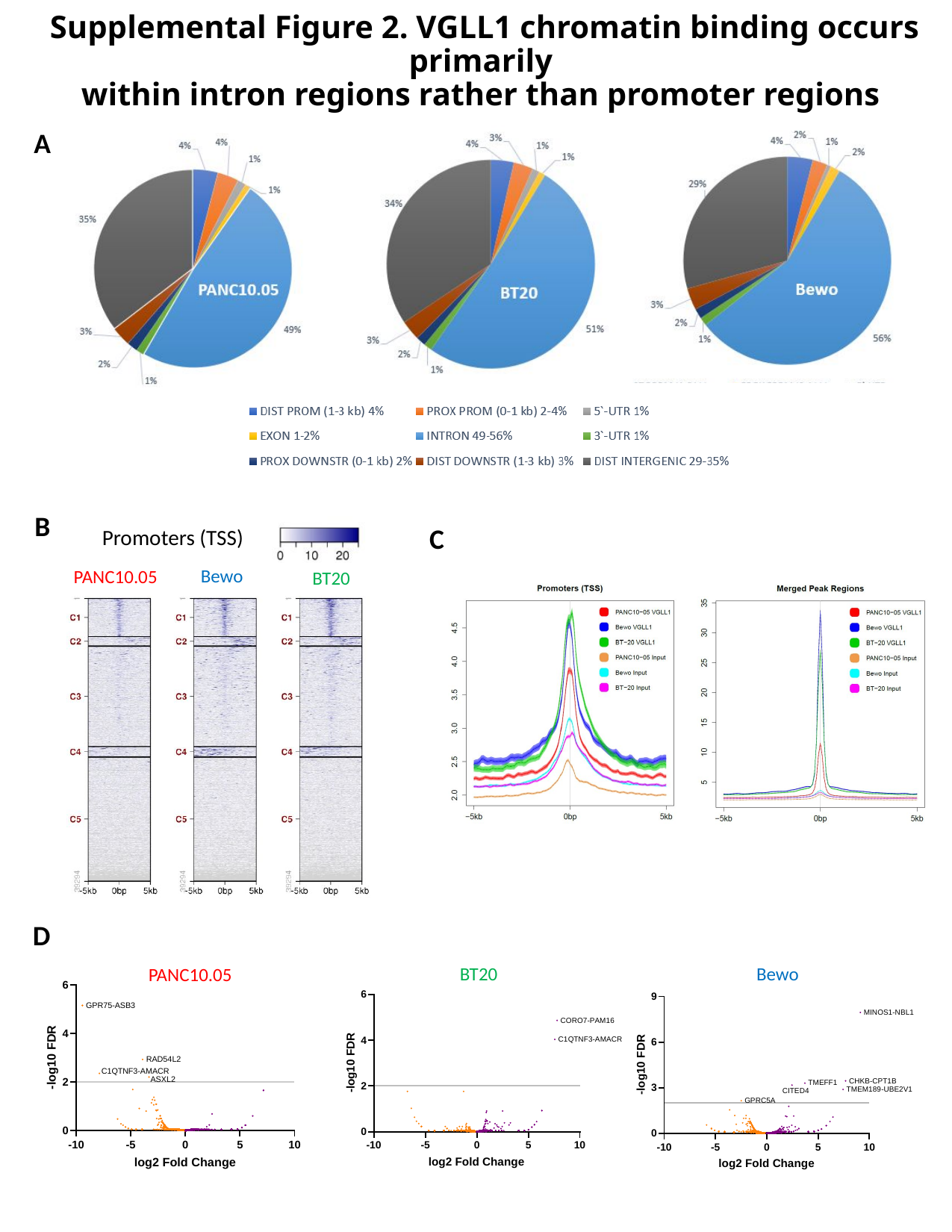

# Supplemental Figure 2. VGLL1 chromatin binding occurs primarily within intron regions rather than promoter regions
A
B
C
Promoters (TSS)
Bewo
PANC10.05
BT20
D
Bewo
BT20
PANC10.05

## Slide 4
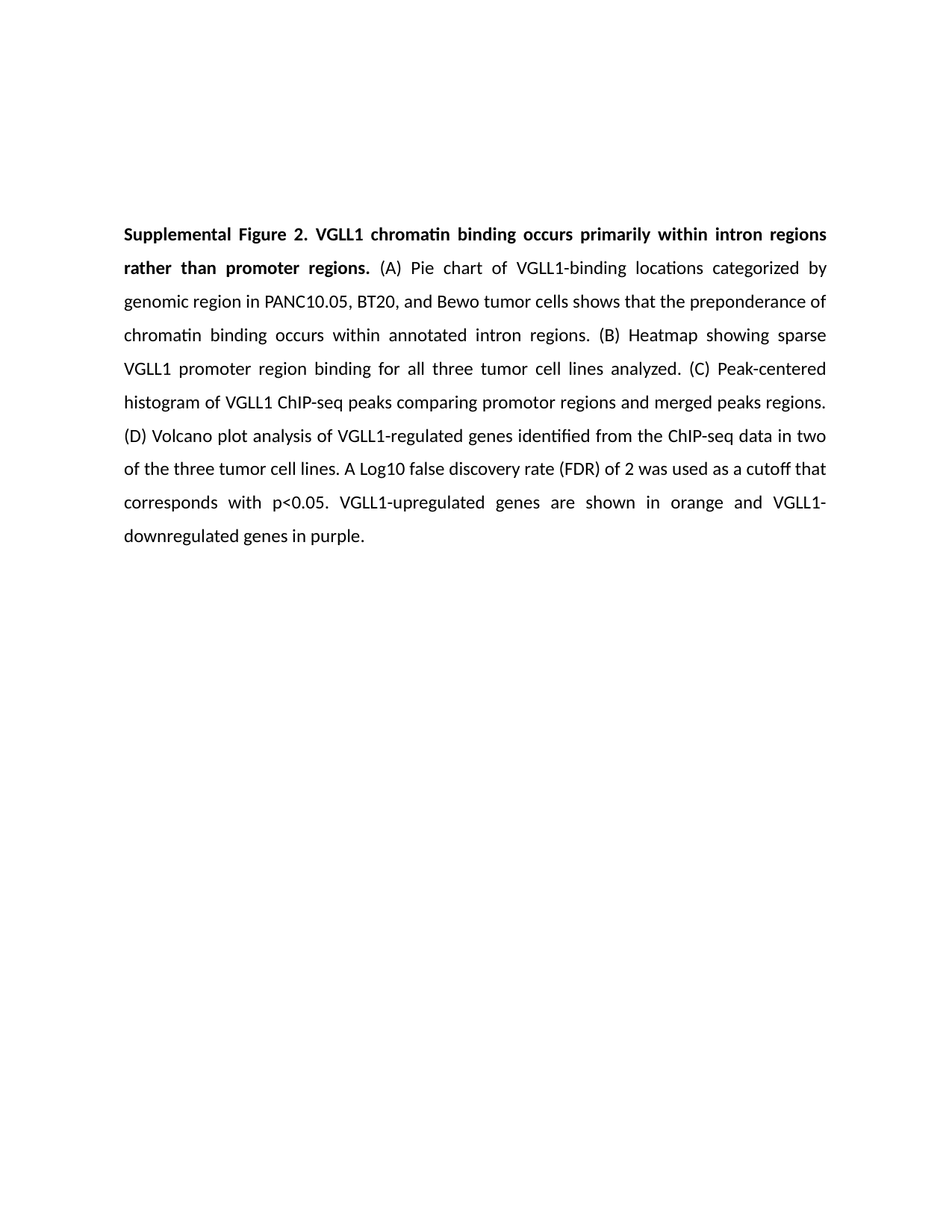

Supplemental Figure 2. VGLL1 chromatin binding occurs primarily within intron regions rather than promoter regions. (A) Pie chart of VGLL1-binding locations categorized by genomic region in PANC10.05, BT20, and Bewo tumor cells shows that the preponderance of chromatin binding occurs within annotated intron regions. (B) Heatmap showing sparse VGLL1 promoter region binding for all three tumor cell lines analyzed. (C) Peak-centered histogram of VGLL1 ChIP-seq peaks comparing promotor regions and merged peaks regions. (D) Volcano plot analysis of VGLL1-regulated genes identified from the ChIP-seq data in two of the three tumor cell lines. A Log10 false discovery rate (FDR) of 2 was used as a cutoff that corresponds with p<0.05. VGLL1-upregulated genes are shown in orange and VGLL1-downregulated genes in purple.

## Slide 5
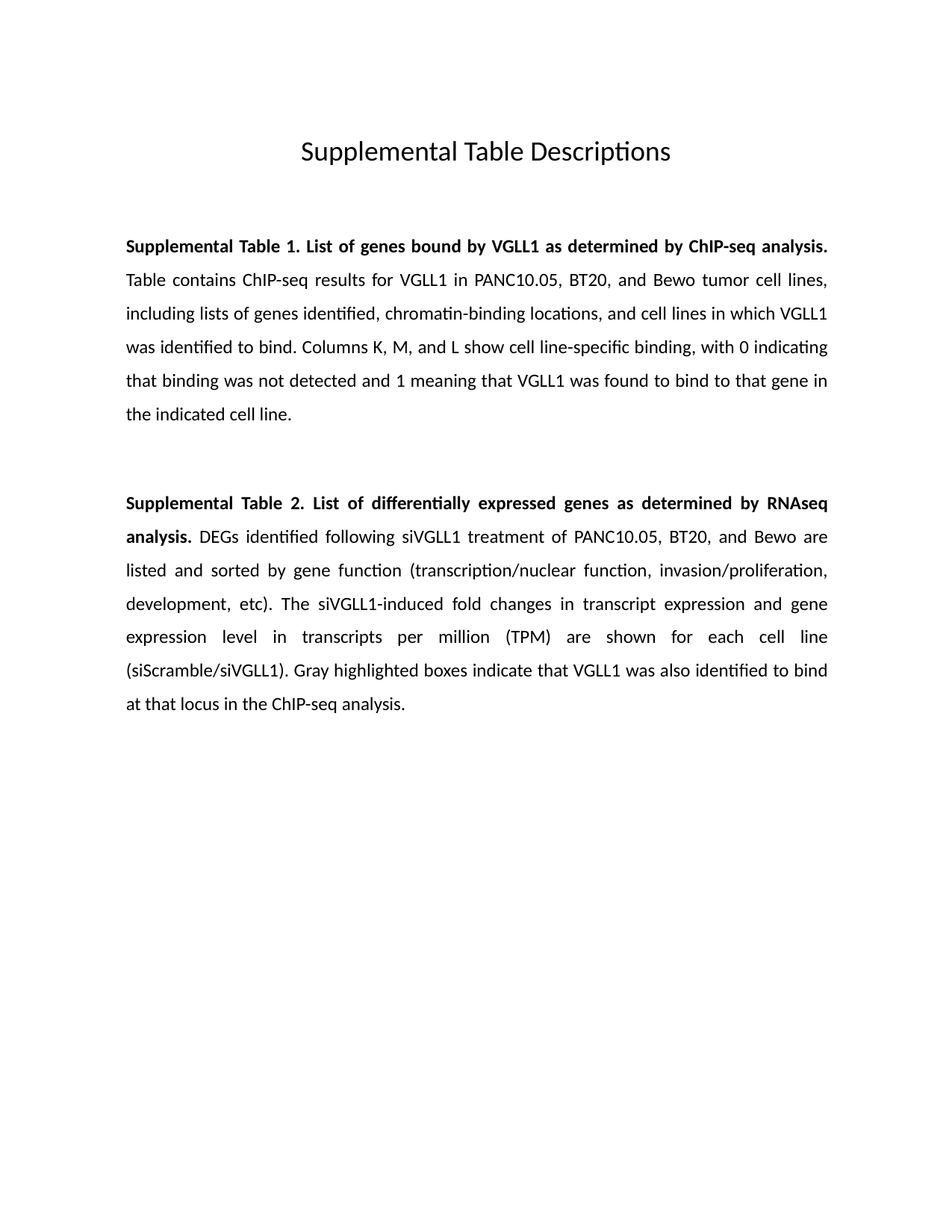

Supplemental Table Descriptions
Supplemental Table 1. List of genes bound by VGLL1 as determined by ChIP-seq analysis. Table contains ChIP-seq results for VGLL1 in PANC10.05, BT20, and Bewo tumor cell lines, including lists of genes identified, chromatin-binding locations, and cell lines in which VGLL1 was identified to bind. Columns K, M, and L show cell line-specific binding, with 0 indicating that binding was not detected and 1 meaning that VGLL1 was found to bind to that gene in the indicated cell line.
Supplemental Table 2. List of differentially expressed genes as determined by RNAseq analysis. DEGs identified following siVGLL1 treatment of PANC10.05, BT20, and Bewo are listed and sorted by gene function (transcription/nuclear function, invasion/proliferation, development, etc). The siVGLL1-induced fold changes in transcript expression and gene expression level in transcripts per million (TPM) are shown for each cell line (siScramble/siVGLL1). Gray highlighted boxes indicate that VGLL1 was also identified to bind at that locus in the ChIP-seq analysis.
